# Addressing Chemotherapy-Induced Peripheral Neuropathy Using Multi-Frequency Vibrometry and Patient-Reported Outcomes

**DOI:** 10.3390/jcm11071862

**Published:** 2022-03-27

**Authors:** Sebastian W. Nielsen, Sanne Lindberg, Christina Halgaard Bruvik Ruhlmann, Lise Eckhoff, Jørn Herrstedt

**Affiliations:** 1 Department of Clinical Oncology and Palliative Care, Zealand University Hospital, 4000 Roskilde, Denmark; sanne.lindberg.01@regionh.dk (S.L.); jherr@regionsjaelland.dk (J.H.); 2 Department of Clinical Research, University of Southern Denmark, 5000 Odense C, Denmark; christina.ruhlmann@rsyd.dk; 3 Department of Oncology R, Odense University Hospital, 5000 Odense C, Denmark; lise.eckhoff@rsyd.dk; 4 Department of Clinical Medicine, Faculty of Health and Medical Sciences, University of Copenhagen, 1165 Copenhagen, Denmark

**Keywords:** chemotherapy-induced peripheral neuropathy, oxaliplatin-induced peripheral neuropathy, paclitaxel-induced peripheral neuropathy, peripheral neuropathy, multi-frequency vibrometry

## Abstract

(1) The study evaluated correlations between multi-frequency vibrometry (MF-V) and the measure of chemotherapy-induced peripheral neuropathy developed by the European Organization for the Research and Treatment of Cancer (CIPN18). (2) Patients with cancer scheduled to undergo treatment with capecitabine and oxaliplatin (CAPOX) or carboplatin and paclitaxel (Carbo-Tax) were recruited in a prospective, observational study with MF-V and the CIPN18 from baseline to one year after end of treatment. (3) The study recruited 31 evaluable patients. All MF-V measurements correlated significantly with the CIPN18 scores (*r* = 0.25–0.48, *p* > 0.003), with a low frequency (32 Hz) from metatarsals showing the best correlation coefficients (0.059 Z-score per CIPN18 point change, *r* = 0.48, CI-95 = [0.32; 0.60], *p* > 0.0001). The largest change in MF-V scores from baseline was seen in low-frequency VPTs taken from metatarsals at 8 Hz three months after end of treatment (from −0.26, CI-95 [−0.85, 0.38] to 1.15, CI-95 [0.53, 1.84]) for patients treated with oxaliplatin and at 32 Hz one year after end of treatment (from 0.09, CI-95 [−0.56, 0.77] to 0.88, CI-95 [0.34, 1.47]) for patients treated with paclitaxel. (4) Low-frequency vibration perception thresholds (8 and 32 Hz) correlated better with CIPN18 scores than high-frequency ones (128 and 250 Hz). If validated, this finding will advance CIPN pathophysiological understanding and inform the development of assessment methods.

## 1. Introduction

Advances in clinical oncology have led to an increasing number of cancer survivors and an awareness of late and long-term treatment effects [1]. Chemotherapy-induced peripheral neuropathy (CIPN) is an important long-term treatment side effect where symptoms severely affect health-related quality of life and inflict an estimated 37–84% of patients after end of treatment [2,3,4]. CIPN has a complex etiology [5] and a continuum of varied symptomatology with no clear diagnostic criteria or cutoffs [6], hampering early screening and management of CIPN. Since CIPN cannot be prevented, it has been managed with treatment alterations, guided by clinician-graded symptom severity such as the National Clinical Institute—Common Terminology Criteria for Adverse Events (NCI-CTCAE) [7]. However, at the time of treatment alteration, many patients will have developed CIPN that most often persists after end of treatment [8,9,10]. This highlights the need for improved, patient-centered, and clinically relevant diagnostic tools with enhanced sensitivity and prognostic capabilities [6,11].

Multi-frequency vibrometry (MF-V) is a novel semiquantitative psychophysiological measurement that captures multiple vibration perception thresholds (VPTs) from tactile non-glabrous skin [12]. Low-frequency MF-V has shown promise in early subclinical detection of diabetic neuropathy, linking measurements to worse glycemic control [13] and also showing a strong association with the risk of developing diabetic ulcers [14]. Studies have already demonstrated a significant correlation between VPTs and CIPN at single frequencies using a tuning fork [15,16,17] or a biothesiometer [18,19,20] in the spectrum of 100–128 Hz in non-tactile locations with some predictive potential [21]. Widening this spectrum to include low-frequency VPTs (8–32 Hz) might provide clinicians with the ability to better discriminate between patients at risk of long-term CIPN and patients with transient symptoms. Low-frequency vibration activates the Meissner’s corpuscles [22], which are severely affected by neurotoxic chemotherapy [23]. These dermal mechanoreceptors account for about 40% of the afferent output from glabrous skin, whereas the Pacinian corpuscles (activated at 100–300 Hz) only innervate about 15% of afferent nerve fibers [22,24]. Based on this, MF-V could improve the sensitivity and clinical usability of CIPN diagnostics by measuring at low, medium, and high frequencies in a 20–30 min operator-independent examination.

This study is aimed at evaluating MF-V as a CIPN assessment by calculating its correlation to CIPN symptoms across multiple assessments before, during, and after treatment with paclitaxel- or oxaliplatin-based chemotherapy. Furthermore, we wanted to explore changes in low-frequency (8 and 32 Hz) and high-frequency (250 Hz) VPTs compared to the standard VPT frequency (128 Hz) used in CIPN. This could potentially elucidate a novel pathophysiological understanding of CIPN, as the physiological processes and structures underlying these sensory modalities differ substantially. 

## 2. Materials and Methods

**Figure 1:**
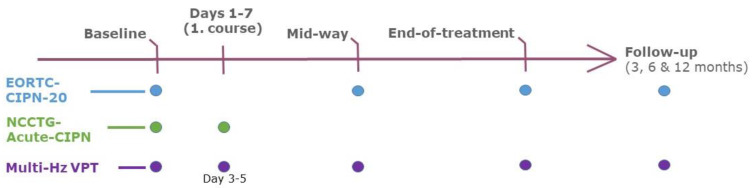
Schematic overview of patient procedures and evaluations across the study period.

We conducted a prospective non-comparative feasibility study capturing repeated measurements of patient-reported outcome measures (PROMs) and MF-V. The study was conducted at the Department of Clinical Oncology and Palliative Care, Zealand University Hospital, Denmark. Eligible patients were aged 18 years or older with a diagnosis of cancer. Patients were required to fulfill criteria for starting chemotherapy and scheduled to undergo at least 4 courses of paclitaxel- or oxaliplatin-based chemotherapy. Patients were excluded if they were unable to complete patient-reported outcome measures (PROMs) or had previously received treatment with neurotoxic chemotherapy (defined as vinca alkaloids, taxanes, or platinum drug derivatives). Patients were evaluated with PROM and MF-V at baseline, during first course of chemotherapy (days 1–7), halfway through treatment (see definition below), and at end of treatment. The intention-to-treat population was defined as all patients with at least one measurement post-baseline. Follow-up was performed 3, 6, and 12 months after end of treatment (Figure 1). Halfway through treatment was defined as after 3 courses of chemotherapy for patients referred for 6 courses of intravenous carboplatin (dose (mg) = (glomerular filtration rate + 25) × 5) and a 3 h infusion of paclitaxel 175 mg/m^2^ (Carbo-Tax) both administered day 0, and after 2 or 4 courses for patients referred for 4 or 8 courses of chemotherapy with intravenous oxaliplatin 130 mg/m^2^ day 0 and oral capecitabine 1000 mg/m^2^ twice daily for fourteen days (CAPOX), respectively. The trial was registered at clinicaltrials.gov before recruitment commenced (NCT04167319).

### 2.1. Measurements

Patient sociodemographic data was captured in a baseline interview. Information was obtained on gender, weight, height, medications, comorbidities, Eastern Cooperative Oncology Group (ECOG) performance status, smoking, and alcohol history. All information was cross-checked with information in the electronic health records. Cancer diagnoses, treatment doses, and treatment changes (dose reductions, delays, or discontinuations) were transcribed from the electronic health records.

CIPN was measured at all evaluations and follow-up visits using the European Organization for the Research and Treatment of Cancer (EORTC)-CIPN20 module [3]. However, we excluded items 19 and 20, calculating the summary score based on items 1–18 (18–72 points), henceforth referred to as the CIPN18 or CIPN18 scores [25,26].

Acute and transient symptoms of paclitaxel- and oxaliplatin-induced peripheral neuropathy were captured with daily questionnaires at baseline until day 6 and a summary questionnaire at day 7 in the first course of chemotherapy. These consisted of single-item PRO measurements developed by the North Central Cancer Treatment Group (NCCTG) for measuring acute paclitaxel [27] and transient symptoms of oxaliplatin-induced peripheral neuropathy [28], as well as the Danish version of the National Cancer Institute-Patient-Reported Outcomes version of the Common Terminology Criteria for Adverse Events (NCI-PRO-CTCAE) General Pain [29]. The NCCTG acute questionnaire items were translated and adapted into Danish using the methodology described by the World Health Organization—Quality of Life Group (WHOQOL) [30] (WHO Translation Report is available on request). 

Multi-frequency vibrometry was performed at baseline. All evaluations and follow-up visits used the VibroSense Meter^®^ II (Vibrosense Dynamics AB, Malmö, Sweden)—a CE-marked Class I medical device licensed to measure vibration perception thresholds (VPTs) between 4 and 500 Hz within a pre-specified skin temperature—and an applied pressure range [12,31]. VPTs were measured at 32, 125, and 250 Hz on the tactile surface of the finger pulp of the index (referred to as DigII-32 Hz, DigII-125 Hz, and DigII-250 Hz) and little finger (DigV-32 Hz, DigV-125 Hz, and DigV-250 Hz) on the patient’s dominant hand, and VPTs at 8, 32, and 125 Hz on the palmar skin of the first (MT-I-8 Hz, MT-I-32 Hz, and MT-I-125 Hz) and fifth metatarsal head (MT-V-8 Hz, MT-V-32 Hz, and MT-V-125 Hz) of the patient’s dominant foot [32]. The procedure is operator-independent, taking between 20 and 30 min to complete. Data were transcribed as age and gender-adjusted z-scores for each frequency and location using a normative population as a reference [12].

### 2.2. Statistical Analysis

Correlation estimates between patient and treatment characteristics and CIPN18 scores at three months and one year after treatment were modeled using linear regression for continuous variables adjusting for age, gender, BMI, and cumulative dose. Logistic regression was used for binary variables, i.e., gender (male/female) and smoking (yes/no). Correlations between baseline neuropathy and CIPN18 scores were adjusted for other baseline covariates, i.e., age, gender, and BMI. Q-Q plots and fitted vs. residuals plots were examined to diagnose any issues with normality or heteroscedasticity in the regression models.

Correlations between CIPN18 scores and VPTs were modeled using a mixed linear model. Repeated measurements were nested within subjects with a random intercept [33]. Model outputs were plotted to ensure equal distribution of fitted values, normality of residuals, and normality of the included random factor. The proportion of variance attributable to subjects as a random factor was estimated by dividing the variance component of the random factor by the total variance to ascertain the appropriateness of including subjects as a random factor. Standardized correlation coefficients (*r* = −1 to 1) and related *p*-values were calculated using the rmcorr package [34]. 

All statistical operations were performed in R-studio (ver. 4.0.3). Mixed linear models were performed using the lme4 and plotted using the ggplot2 packages. Missing data were assumed missing-at-random and omitted from analyses. *p*-values were adjusted for the false discovery rate [35], treating patient- and treatment-related analyses and repeated measurement correlation analyses as two separate hypotheses. The significance level was 0.05.

The method of multi-frequency tactilometry has not been investigated in this population and setting; as such, there are no estimates for sample size justification. However, 12 patients in each group will provide a good estimate of variance and mean for this continuous outcome [36]. Expecting a 15% drop out rate, we will have to recruit 30 evaluable patients, which is in concurrence with prior evaluations for sample sizes in pilot studies [37].

## 3. Results

The patients were recruited from 22 November 2019 to 2 July 2020, and the last patient/last follow-up was 11 November 2021. The rate of missing evaluations was 12% and was primarily caused by COVID-19 restrictions, which restricted the accessibility of patients to research activities and the movement of personnel during the study period (27% of all missing evaluations were attained during the first lockdown period from 11 March to 15 April 2020).

### 3.1. Patient and Treatment Characteristics

A total of 32 patients were recruited. Two died before reaching the final follow-up (one from neutropenic sepsis, and one from relapse), and one patient chose to leave the study before completing any follow-up visits. (Table 1). Finally, 31 patients were included in the analyses. Patients were most often referred for adjuvant treatment of colon (31%) or ovarian cancer (41%) with stage 3 (65%) or 4 (22%) disease. Women constituted 84% of the cohort, and the patients’ performance status was 0 (77%) or 1 (23%). Type 2 diabetes was the most common comorbidity (12%), and 44% of patients had at least one comorbidity at baseline. Patients treated with Carbo-Tax received an average of four cycles of paclitaxel with a cumulative dose of 618 mg/m^2^ (Standard Error (S.E) 68 mg/m^2^), and patients treated with CAPOX received an average of four cycles of oxaliplatin with a cumulative dose of 436 mg/m^2^ (S.E 54 mg/m^2^). In total, 16 patients experienced dose reduction of the neurotoxic component, where CIPN was cited as the main reason in 13 cases. Discontinuation of the neurotoxic component was experienced by 21 patients: here, CIPN was the main reason in 10 cases, whereas paclitaxel infusion reaction and relapse accounted for four and two cases, respectively (Table 1 and Supplementary Materials). The residual reasons were chemotherapy-induced nausea and vomiting (*n* = 1), recurrent infection (*n* = 1), thrombocytopenia (*n* = 1), worsening performance score (*n* = 1), and patient choice (*n* = 1). No significant correlations were found between patient- and treatment-related variables, or NCCTG acute PRO scores and CIPN18 scores at three months and 1-year follow-up (Table 2). 

### 3.2. CIPN18

**Figure 2:**
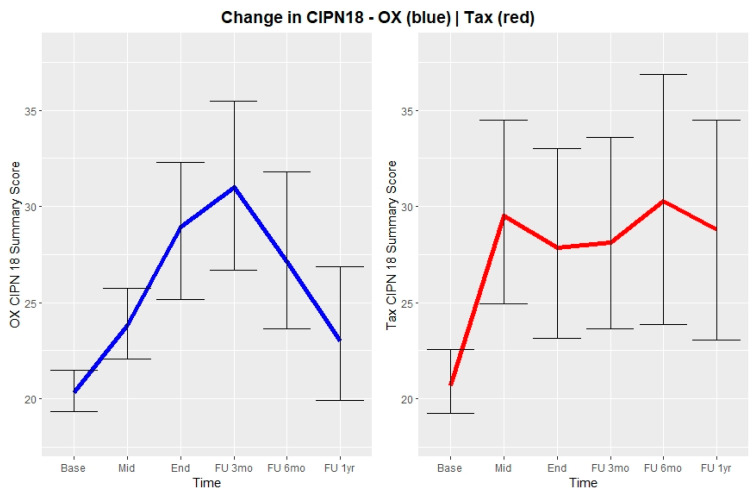
Development in patient mean CIPN18 scores from baseline to follow-up 1 year after end of treatment with corresponding 95% confidence intervals displayed as error bars. Left, blue: Depicts patients in CAPOX treatment. Right, red: Depicts patients in Carbo-Tax treatment.

Mean CIPN18 scores increased significantly for patients treated with Carbo-Tax or CAPOX, reaching peak mean scores after three cycles for the Carbo-Tax group (29 points (Interquartile range (IQR) = 15)) and three months after end of treatment for patients treated with CAPOX (31 points (IQR = 10)). Although the mean score among patients treated with Carbo-Tax remained high (28 points (IQR = 16)) at the last follow-up one year after end of treatment, the mean score among patients treated with CAPOX improved markedly, showing near normalization at the last follow-up, one year after end of treatment (21 points (IQR = 4) (Figure 2).

### 3.3. Vibrations Perceptions Thresholds 

**Figure 3:**
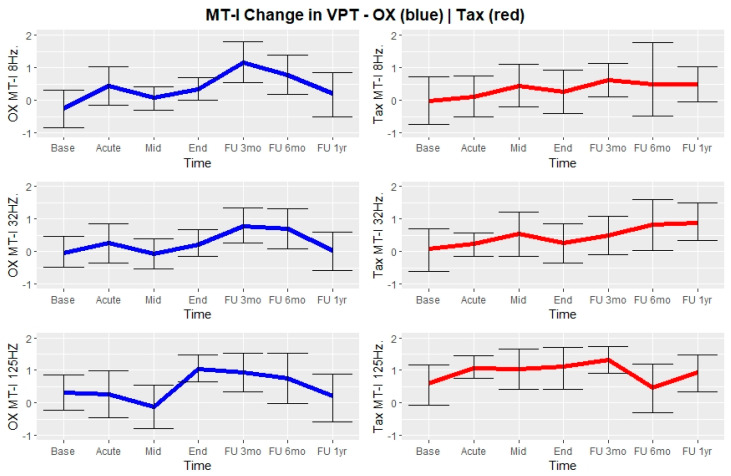
Development of mean VPT Z-scores taken from patient’s dominant metatarsal I (palmar surface) from baseline to 1 year after end of treatment including the acute measurement taken once between day 3 and 5 in the 1 course of chemotherapy. Error bars represent 95% Confidence intervals of mean VPT Z-scores. Left, blue: Depicts patients in CAPOX treatment. Right, red: Depicts patients in Carbo-Tax treatment.

In general, a change in the 12 VPTs measurements showed a steady, progressive increase in mean z-scores (worsening of vibration sense) across time for almost all frequencies in both hands and feet and subsequently returned to baseline for patients treated with oxaliplatin and to a lesser degree for patients treated with paclitaxel. The largest change from baseline for patients treated with oxaliplatin was at MT-I-8 Hz three months after end of treatment (from −0.26, CI-95 = (−0.85, 0.38) to 1.15, CI-95 = (0.53, 1.84)), and for patients treated with paclitaxel at MT-I-32 Hz 1 year after end of treatment (from 0.09, CI-95 = (−0.56, 0.77) to 0.88, CI-95 = (0.34, 1.47), (Figure 3 and Supplementary Material for additional figures of VPT change over time by location, these are Dig-II (Figure S1), Dig-V (Figure S2) and MT-V (Figure S3). 

Acute VPT measurements between days 3 and 5 after the first course of chemotherapy showed the largest mean change from baseline in MT-I-8 Hz in patients receiving CAPOX (0.7, CI-95 (−0.23, 1.62)) and in Dig-V-32 Hz (0.64, CI-95 (−0.26, 1.53)) for patients receiving Carbo-Tax (Figure 3 and Supplementary Materials).

In multiple regression analyses, baseline high-frequency VPT scores significantly correlated with CIPN18 scores at three months (DigII-125 Hz and MT-V-125 Hz) and one-year follow-up (DigII-125 Hz, Dig-II-250 Hz) after end of treatment in patients receiving oxaliplatin, but not after adjusting for multiple comparisons (Table 2). The largest magnitude of the correlation between baseline VPT scores and long-term CIPN18 was seen for patients receiving paclitaxel with 8.37 points at three months per baseline z-score and 10.57 points per baseline z-score at 1 year. 

### 3.4. CIPN18 in Relation to VPTs

All correlations between the CIPN18 score and MF-V frequencies were significant after adjusting for multiple comparisons and were of a weak strength, r = [0.25; 0.48]. The weakest correlations were found between CIPN18 and VPT at DigII-32 Hz and the strongest between CIPN18 and VPT at MT-I-32 Hz (see Table 3).

## 4. Discussion

This feasibility study investigated CIPN development in patients treated with oxaliplatin or paclitaxel-based chemotherapy. Baseline measurements of PRO and MF-V were repeated during and after treatment with neurotoxic chemotherapy and patients were followed for one year. This is the first study to explore differential VPT scores including a low range of frequencies (8–32 Hz) in oxaliplatin- and paclitaxel-treated patients over time.

In essence, MF-V correlated weakly but significantly with CIPN18 scores, with low-frequency VPTs yielding better correlations than high-frequency VPTs. Other studies have found similar significant correlations between VPTs and CIPN outcomes [16,17,18,19,20], yet this study demonstrated that lower-frequency VPTs (8–32 Hz) in tactile locations correlate better and with greater magnitude than higher-frequency VPTs in non-tactile locations (125–250 Hz). This finding could have several implications for future studies of CIPN. MF-V could help the development of a clinically relevant, predictive CIPN measure. An earlier study showed that the difference between CTCAE grades 0 and 2 was 14.1 points for patients treated with paclitaxel and 8.3 points for patients treated with oxaliplatin [25]. Even with large overlaps between grades, finding large correlation coefficients in this study between baseline MF-V and follow-up CIPN18 scores at three months and one year after end of treatment (8–10 points) could hold the potential for CIPN risk ascertainment in conjunction with other risk factors. In regards to other clinically relevant outcomes, a recent study found a greater involvement of low-frequency (30 Hz) VPTs compared to high-frequency VPTs (200 Hz) in patients with diabetic peripheral neuropathy [38] and earlier MF-V findings in diabetic patients have demonstrated a correlation between low-frequency MF-V and clinically important outcomes such as the risk of diabetic foot ulcers and glycemic control in diabetic children [13,14,39]. However, correlation does not necessarily mean good sensitivity and specificity in clinical prediction [21]. CIPN is a term covering many drug-specific neuropathies developing in patients given the interaction of many different risk factors [40,41]. With this in mind, it is unlikely that baseline MF-V scores alone can discriminate perfectly between all patients with transient symptoms and patients developing long-term symptoms of CIPN in every particular type of neuropathy. Here, several baseline risk factors such as baseline blood samples (hemoglobin, micronutrients, neutrophil-to-lymphocyte ratio, etc.) and patient risk factors [42] could be analyzed together with semiquantitative measures of high correlation with CIPN outcomes (such as MF-V VPTs) to produce a more accurate prediction for each patient by following prior examples where machine learning has been utilized to predict the risk of vincrinstine-induced peripheral neuropathy [43] and diabetic polyneuropathy [44].

In addition to improved CIPN diagnostics, the finding of the involvement of lower-frequency vibration in CIPN is important as there are central underlying histological and neurophysiological distinctions between sensory signaling emerging from low-threshold mechanoreceptors (LTMRs) associated with the end-organ structures Pacinian corpuscles (Aβ RA-II LTMR) and Meissner’s corpuscles (Aβ RA-I LTMR), activating optimally at vibrations around 150–200 Hz and 40–60 Hz, respectively [45]. Both Pacinian and Meissner’s corpuscles are rapidly adapting low-threshold mechanoreceptors (RA-LTMRs) and signaling is conveyed through Aβ-fibers, which means that nerve-conduction studies are not able to distinguish between signals arising from these sensory end-organ structures. Meissner’s corpuscles are associated with grip-control and object manipulation, sharing a close anatomical relationship with Merkel cells associated with detecting object points and edges similar to rods and cones in the retina, whereas Pacinian corpuscles are associated with vibrotactile feedback across larger receptive fields [45]. Meissner’s corpuscles are severely affected by neurotoxic chemotherapy, showing a 50% decrease after treatment with paclitaxel and a total decay after treatment with bortezomib compared to healthy controls [46,47]. Although lower extremities are often more severely affected in persistent, long-term CIPN, impairment of upper-limb sensorimotor function in CIPN is also significantly affected [48]. The results from the present study may pinpoint this impairment of upper extremity function (grip and object manipulation) further to a functional loss of Meissner’s corpuscles rather than Pacinian corpuscles. This has implications for CIPN pathophysiology as greater comparative (Meissner’s vs. Pacinian) understanding of damaging mechanisms may provide further reasoning for preventive strategies. 

This study is limited by its small, explorative sample size. Several studies have found a relationship between CIPN and cumulative doses of neurotoxic chemotherapy, diabetes, age, and gender [7]. This was not found in the present study, but this is most likely due to the restricted power in a multivariate analysis of patient- and treatment-related factors and long-term CIPN18 scores given the small sample size. VPT change over time showed several non-overlapping confidence intervals of the VPT means over time compared to baseline in both groups. These were not submitted to hypothesis testing as the amount of *p*-values from such an analysis would be 40, requiring tests to obtain at least a *p*-value of 0.00125 to be considered significant in this study. In this perspective, the most marked change from baseline was seen in MT-I-8 Hz in patients treated with oxaliplatin, which achieved a *p*-value of 0.005. However, the findings show promise and have been carried forward as a priori hypotheses in an ongoing study (CINCAN-2) investigating the potential preventive effect of cannabidiol on CIPN.

## 5. Conclusions

This study found that low-frequency VPTs in the range of 8–32 Hz correlated better in strength and magnitude with CIPN18 scores than high-frequency ones (125–250 Hz) VPTs. Furthermore, the largest change in VPTs over time was found in low frequencies in the metatarsals at 8 Hz and 32 Hz for patients treated with CAPOX and Carbo-Tax, respectively. Based on this, it is possible that low-frequency VPTs may be more severely affected by neurotoxic chemotherapy than high-frequency VPTs. If validated, these findings hold importance for CIPN pathophsyiological understanding and the future development of assessments for risk stratification. We seek to confirm these findings in the CINCAN-2 trial (National Clinical Trials identifier: 04582591).

## Figures and Tables

**Table 1:** Patient characteristics.

		Overall	Oxaliplatin	Paclitaxel
	N (%)	Mean	Mean (Range)	Mean (Range)
Age and Gender				
Overall	31	58		
Women	26 (84)	59	58 (46–72)	60 (47–77)
Men	5 (16)	51	51 (42–59)	n/a
BMI				
Overall	31	26.5	25.3 (20.5–33.7)	27.1 (16.1–41.1)
Comorbidities				
None	18 (56)		10	8
Diabetes	3 (9)		1	2
Hypertension	2 (6)		1	1
COPD	2 (6)		0	2
Arthritis	3 (9)		0	3
Alcohol overconsumption (≥14 units/week)	1 (3)		1	0
Other	6 (19)		2	4
With polymorbidity (≥2)	3 (9)		0	3
With polypharmacy (≥5)	4 (12)		1	3
Smoking				
Yes	9 (29)		3	6
No (+former)	22 (71)		12	10
Alcohol Status				
No (abstains)	4 (13)		0	4
Yes	27 (89)		15	12
Cancer Diagnoses				
Rectal	4 (13)		4	
Colon	10 (31)		10	
Appendix	1 (3)		1	
Endometrial	2 (6)			2
Ovarian and Fallopian tube	14 (44)			14
Stage—Colorectal cancers				
III	12 (39)		12	
IV	4 (13)		4	
Stage—Gynecological cancers				
I	2 (6)			2
II	2 (6)			2
III	9 (29)			9
IV	3 (9)			3
Performance status (ECOG)				
0	24 (77)		12	12
1	7 (23)		3	4

**Table 2:** Linear correlations between patient- and treatment-related factors, baseline VPT scores, and CIPN18 scores at 3 and 12 months for patients treated with oxaliplatin or paclitaxel. Adjusted for age, gender, BMI, and cumulative dose.

	Oxaliplatin		Paclitaxel	
	3 Months after Treatment (*n* = 13)	12 Months after Treatment (*n* = 14)	3 Months after Treatment (*n* = 14)	12 Months after Treatment (*n* = 15)
	Beta-Coefficient	Beta-Coefficient	Beta-Coefficient	Beta-Coefficient
Age	0.43	0.12	−0.06	0.18
Gender (Ref.: Male)	0.62	−1.21	N/a	N/a
BMI	0.42	−0.12	0.18	−0.13
Smoking (Active)	4.83	−3.27	1.62	−1.86
Alcohol weekly	0.03	−0.22	−0.36	0.09
No. medications (0–9)	−2.08	−1.43	1.63	1.75
Cumulative dose (/100 mg)	0.20	0.03	−0.10	0.02
Baseline z-scores compared to follow-up CIPN18 score				
Dig-II-32 Hz	−1.00	−3.47	−4.56	−3.03
Dig-II-125 Hz	4.23	3.76 *	5.02	0.31
Dig-II-250 Hz	4.86 *	3.13 *	8.37	10.52
Dig-V-32 Hz	0.39	0.72	0.39	0.64
Dig-V-125 Hz	2.04	2.74	2.02	−1.74
Dig-V-250 Hz	2.99	2.18	−0.02	−2.61
MT-I-8 Hz	3.26	1.30	−0.92	2.78
MT-I-32 Hz	2.48	1.11	0.83	2.91
MT-I-125 Hz	2.20	0.66	2.80	3.61
MT-V-8 Hz	0.87	−1.46	−1.10	1.14
MT-V-32 HZ	3.21	0.84	0.32	1.82
MT-V-125 Hz	8.44 *	2.88	0.95	1.09
NCCTG Acute Neuropathy Scores compared to follow-up CIPN18 score				
TAPS Sum Score			1.94	1.63
Acute CIPN Sum Score	−1.16	−0.36		

* Only significant before adjusting for multiple comparisons, *p*-value < 0.05. Note that regression models on baseline z-scores and NCCTG scores were not adjusted for cumulative dose. Abbreviations: NCCTG, North Central Cancer Treatment Group; Dig, Digitus; MT, Metatarsal.

**Table 3:** Estimated magnitude of linear relationship and correlation between MF-V frequencies and patient-reported outcomes (CIPN18).

	CIPN18 Score			
	Coefficient ± S.E.	*r*	CI-95	*p*
Dig-II-32 Hz	0.019 ± 0.007	0.25	(0.08, 0.45)	0.003
Dig-II-125 Hz	0.045 ± 0.008	0.43	(0.28, 0.57)	<0.0001
Dig-II-250 Hz	0.042 ± 0.009	0.35	(0.18, 0.49)	<0.0001
Dig-V-32 Hz	0.034 ± 0.008	0.35	(0.18, 0.49)	<0.0001
Dig-V-125 Hz	0.041 ± 0.007	0.40	(0.24, 0.54)	<0.0001
Dig-V-250 Hz	0.051 ± 0.010	0.37	(0.20, 0.50)	<0.0001
MT-I-8 Hz	0.058 ± 0.009	0.45	(0.29, 0.58)	<0.0001
MT-I-32 Hz	0.059 ± 0.009	0.48	(0.32, 0.60)	<0.0001
MT-I-125 Hz	0.046 ± 0.011	0.32	(0.14, 0.47)	0.003
MT-V-8 Hz	0.051 ± 0.009	0.38	(0.22, 0.52)	<0.0001
MT-V-32 Hz	0.045 ± 0.009	0.36	(0.19, 0.50)	<0.0001
MT-V-125 Hz	0.051 ± 0.008	0.45	(0.29, 0.58)	<0.0001

*p*-values adjusted for multiple comparisons, significance level < 0.05. Coefficients are z-score change per one point CIPN18 score change.

## Data Availability

The datasets can be made available from the corresponding author upon reasonable request.

## Supplementary Materials

The following supporting information can be downloaded at: https://www.mdpi.com/article/10.3390/jcm11071862/s1, Table S1: Treatment characteristics; Figure S1: Development of mean VPT Z-scores taken from patient’ dominant index finger from baseline to 1-year after end-of-treatment including the acute measurement taken once between day 3–5 in the 1. Course of chemotherapy. Errorbars represent 95% Confindence intervals of mean VPT Z-scores. Left-Blue: Depicts patients in CAPOX treatment. Right-Red: Depicts patients in Carbo-Tax treatment; Figure S2: Development of mean VPT Z-scores taken from patient’ dominant little finger from baseline to 1-year after end-of-treatment including the acute measurement taken once between day 3–5 in the 1. Course of chemotherapy. Errorbars represent 95% Confindence intervals of mean VPT Z-scores. Left-Blue: Depicts patients in CAPOX treatment. Right-Red: Depicts patients in Carbo-Tax treatment; Figure S3: Development of mean VPT Z-scores taken from patient’ dominant metatarsal V (palmar surface) from baseline to 1-year after end-of-treatment including the acute measurement taken once between day 3–5 in the 1. Course of chemotherapy. Errorbars represent 95% Confindence intervals of mean VPT Z-scores. Left-Blue: Depicts patients in CAPOX treatment. Right-Red: Depicts patients in Carbo-Tax treatment.
