# Supplementary material for: Addressing Chemotherapy-Induced Peripheral Neuropathy Using Multi-Frequency Vibrometry and Patient-Reported Outcomes
Source: J Clin Med. 2022 Mar 27;11(7):1862. doi: 10.3390/jcm11071862 (PMC8999713; doi:10.3390/jcm11071862)
Supplement: Supplementary file 1 [file jcm-11-01862-s001.zip › jcm-1629339-supplementary.pdf]

Supplementary Table S1: Treatment characteristics

**Courses and dose**	N	Mean	Range	S.E
Oxaliplatin	16			
- Number of courses given	59	4	1-8	
- Cumulative dose (mg/m^2)		436	129-1023	54
Paclitaxel	15			
- Number of courses given	60	4	1-8*	
- Cumulative dose (mg/m^2)		618	172-1063	69
**Dose delay and discontinuation**	**N**			**%**
Dose delays	14 / 31			45
Main reason:				
- Neutropenia	6			
- Thrombocytopenia	3			
- CIPN	1			
- CINV	1			
- Fatigue	1			
- Unrelated to CT	2			
Dose reduction	16 / 31			52
Main reason:				
- CIPN	13			
- CINV	2			
- Thrombocytopenia	1			
Discontinuation of neurotoxic chemotherapy	21 / 31			68
Main Reason:				
- CIPN	10			
- Taxol Reaction	4			
- Relapse	2			
- Other reasons	5			

* One patient relapsed and was reinduced with Carbo-Tax before end-of-study

Supplementary Figure S1

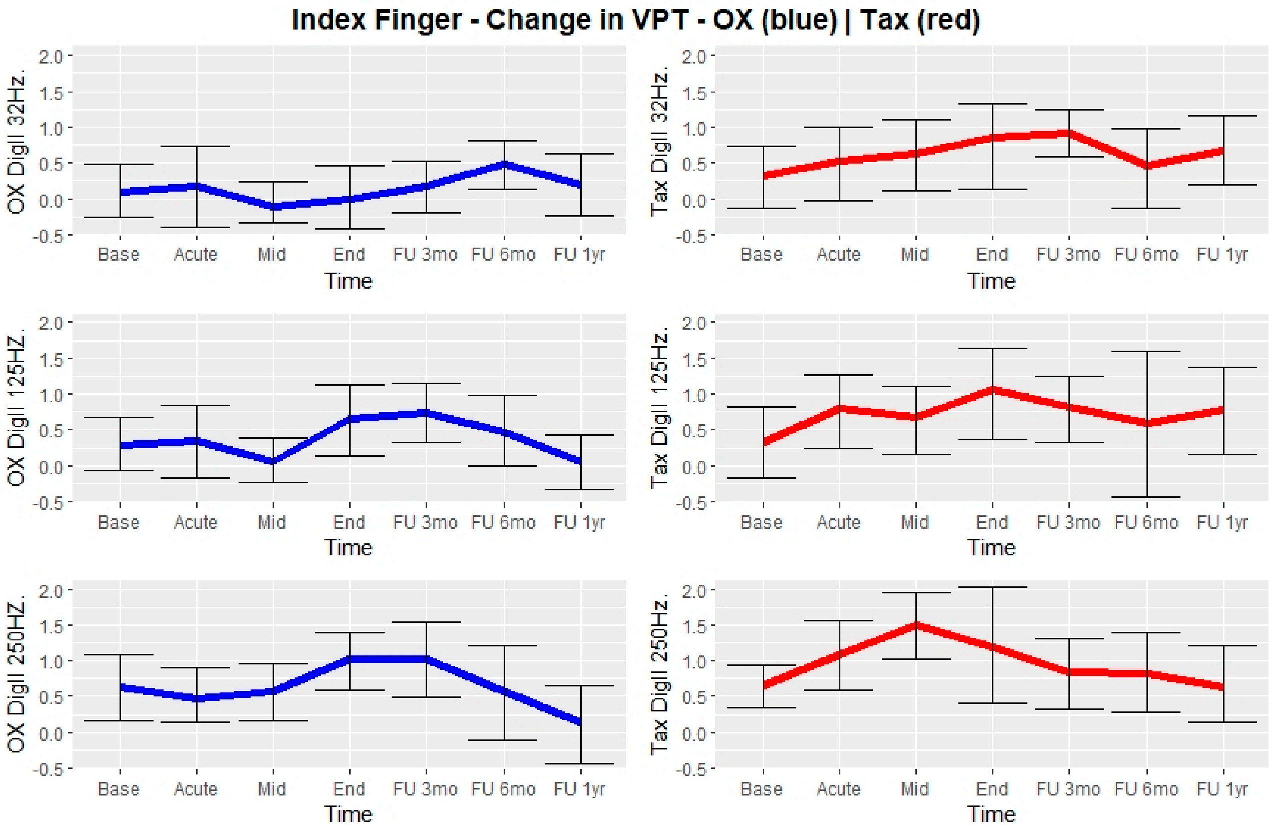

Suppl.Fig S1 *Development of mean VPT Z-scores taken from patient' dominant index finger from baseline to 1-year after end-of-treatment including the acute measurement taken once between day 3-5 in the 1. Course of chemotherapy. Errorbars represent 95% Confindence intervals of mean VPT Z-scores. Left-Blue: Depicts patients in CAPOX treatment. Right-Red: Depicts patients in Carbo-Tax treatment.*

Supplementary Figure S2

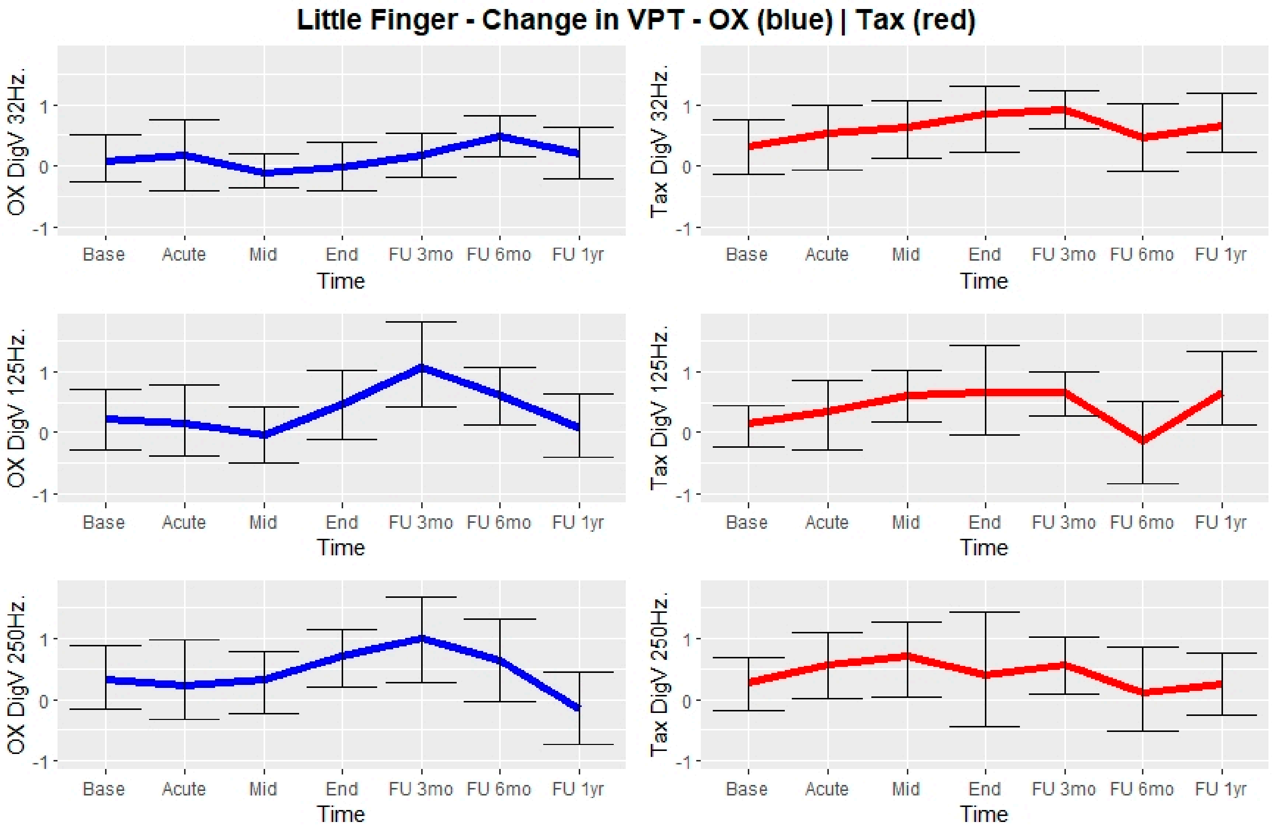

***Suppl.Fig S2** Development of mean VPT Z-scores taken from patient' dominant little finger from baseline to 1-year after end-of-treatment including the acute measurement taken once between day 3-5 in the 1. Course of chemotherapy. Errorbars represent 95% Confindence intervals of mean VPT Z-scores. Left-Blue: Depicts patients in CAPOX treatment. Right-Red: Depicts patients in Carbo-Tax treatment.*

Supplementary Figure S3

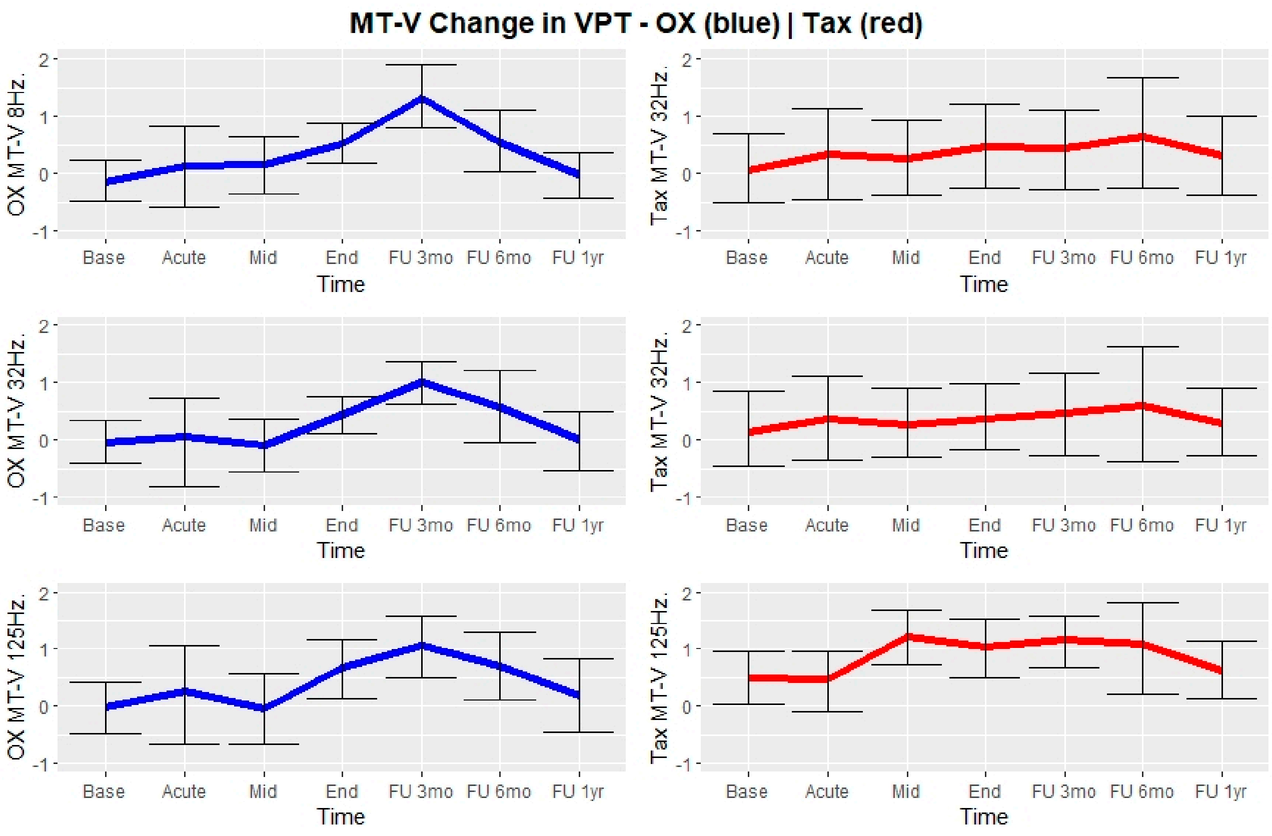

Suppl.Fig S3 *Development of mean VPT Z-scores taken from patient' dominant metatarsal V (palmar surface) from baseline to 1-year after end-of-treatment including the acute measurement taken once between day 3-5 in the 1. Course of chemotherapy. Errorbars represent 95% Confindence intervals of mean VPT Z-scores. Left-Blue: Depicts patients in CAPOX treatment. Right-Red: Depicts patients in Carbo-Tax treatment.*
